# Anecdote of M. Orfila

**Published:** 1849-04

**Authors:** 


ANECDOTE OF M. ORFILA.

Orfila, the eminent chemist, had long been Dean of the Faculty of the School of Medicine, in Paris, when the revolution of February broke out. A royalist in principle, a decided partisan, ardently attached to the person and the cause of Louis Phillipe, the favorite of Guizot, it was natural when the reins of government passed into other hands that he should be looked upon with an evil eye. The enthusiastic young republicans in the medical school were inflamed against him; he was disliked by most of the members of the Provisional Government. In his office of Dean he had handled much of the public money, and was charged with having applied large sums to his own private use. He was removed, and M. Bouillaud placed at the head of the Faculty. Orfila asked that an inquiry should be made into his administration, and the result of a patient and thorough investigation was his triumphant acquittal,

Bouillaud, his bitterest enemy, concurring in the justice of the decision. The following anecdote was related to us while in Paris, and as it is characteristic of this remarkable man, whose history has acquired new interest from these events, we have concluded to repeat it here.

We were seated with a classmate one morning in the cafe, which immediately fronts the *Ecole Pratique*, drinking chocolate and reading the morning journals, when Orfila passed hurriedly by the window, and entering the tall, arched gateway, was soon lost to our view. There, said my friend, a young Frenchman, is one of the most remarkable men in Paris, the firmest, the sternest, the most inflexible Spaniard I ever saw. Some years ago, he continued, through his influence, aided by that of a few others, M. Royer Collard was introduced into the School of Medicine, in opposition to the sense of the profession, and to the feelings of the class. Indeed, so unpopular with the latter was the appointment that they resolved to hiss Royer Collard down whenever he should attempt to lecture before them. Not long after he had been vested with the professorial gown, Royer Collard arose in the Amphitheatre of the School of Medicine to deliver his introductory lecture. The room was crowded to overflowing; the door ways were blocked up; even the passages were filled, so great was the curiosity and exasperation of the students. The newly created professor had scarcely uttered the word *Messieurs*, when the hisses began and soon became so loud and universal that he was obliged to desist. He sat down, and the hissing and tumult gradually subsided. He rose the second time; but again the deafening noise obliged him to refrain. He sent for Orfila, Dean of the Faculty. Orfila entered stealthily at a side door, and, shielded from the view of the class by a black-board, but visible to Royer Collard, beckoned him to resume his discourse. "*Messieurs*" was again uttered, but had hardly passed his lips when the students, furious at his obstinacy, fairly yelled out; the tumult was greater than ever. The lecturer, his face blanched with rage, again sat down, and the uproar soon subsided. Orfila stepped from behind his cover, walked forward in front of the class, folded his arms across his breast, and, turning to the insulted professor, said in a clear, distinct, firm voice, "*Monsieur Royer Collard, continuez.*" Royer Collard, obedient to the command, stood up, evidently very much inspirited by the presence of the Dean, and, throwing as much confidence as he was master of into his tones, attempted to proceed with his lecture. The house rang with hisses, yells, and groans. Orfila turned slowly round and cast his eyes over the multitude. The noise at once ceased. Then raising his voice, he again repeated, "*Monsieur Royer Collard, continuez.*" And the persevering professor once more resumed his lecture. '*Messieurs,*' he began; but a tempest of hisses, mingled with groans, again obliged him to sit down. Orfila singled out a student who had been prominent in the disturbances, cleared the railing which separated them at a single bound, seized him by the nape of the neck, pushed him to the door, reached the stairway, and kicked him to the bottom of the stairs. Returning, he resumed his former position, and in the same firm, clear tone said, "*Monsieur Royer Collard, continuez.*" Royer Collard rose once more to his feet, commenced his discourse, and such was the effect of the Dean's movement, that to the close of his long lecture a pin might have been heard to fall in any part of that vast assemblage. We rejoice that this stern old philosopher has passed unharmed through the fiery ordeal to which he has been subjected. His fame belongs to the world, and we should grieve at any occurrence that would abate its luster. D. W. Y.

